# Study on the extraction and purification of glycoprotein from the yellow seahorse, *Hippocampus kuda* Bleeker

**DOI:** 10.1002/fsn3.219

**Published:** 2015-03-23

**Authors:** Yuting Su, Yongjian Xu

**Affiliations:** School of Marine Sciences, Ningbo University/Key Laboratory for Applied Marine Biotechnology of Ministry of EducationNingbo, 315211, China

**Keywords:** Extraction, glycoprotein, *Hippocampus kuda* Bleeker, purification

## Abstract

The optimum parameters of extraction for glycoprotein from seahorse were examined and determined by Box-Behnken combined with ultrasonic extraction technology. Column chromatography of glycoprotein was used for further purification. The optimal extraction conditions of seahorse glycoprotein were extracting time 4.3 h, salt concentration 0.08 mol/L, extracting temperature 73°C, raw material, and water ratio 1:6. At the optimal conditions, the yield of saccharide reached to 1.123%, and the yield of protein reached to 5.898%. For purifying the crude glycoprotein, the stage renounces of DEAE-52 column chromatography were done, respectively, with 0.05, 0.1, 0.5 mol/L NaHCO_3_ solution, and further purification was done with Sephadex G-100 column chromatography. Finally, two pieces of seahorse glycoprotein were obtained by the column chromatography, that is, HG-11 and HG-21. The saccharide content was 56.7975% and 39.479%, the protein content was 30.5475% and 51.747%, respectively.

## Introduction

The seahorse, a traditional resource as medicine and seafood, is famous as the reputation of “Northern ginseng, Southern seahorse” in China. There are six species of the seahorses were recorded in ancient Chinese medicine books with the same pharmacological functions (Xu 2007), and also some special compositions were detected by modern pharmacochemistry (Xu et al. 2002, 2014; Nie 2008). Modern medicine derived from marine lives has special and unique chemical composition and structure with a high specific activity and efficacy, which is different from those of terrestrial organisms (Yang 2009; Xie and Nie 2010). Glycoprotein is such a class of active substance. In recent years, marine medicine research has discovered that glycoprotein has excellent effects about heath care, such as antitumor (Huang et al. 2009), antioxidation (Liu et al. 2008; Li 2011), anti-inflammatory (Yang et al. 2005), antifatigue (Lin et al. 2009), and improving the immune system (Zhang and Lei 2008). The glycoprotein is involved in the mechanism of the directed homing of cell, glycoprotein hormones, and toxins. Through selectively concentrate in the lesions, it can significantly improve efficacy and reduce adverse reactions without affecting the growth of normal tissue and the function of immune system. It has been shown that more than 80% of protein are glycoprotein (Li 2011; Lu 2013), including a plurality of enzymes, hormones, toxins, carrier proteins, immunoglobulins, structural protein, lectins, receptors, other components of mucus, even the “pure” polysaccharides, which had been considered in the past, also contain a small amount of covalently binding protein. The structure of glycoprotein is complex and diverse. The studies on glycoprotein have concentrated on the physiological and pharmacological aspects of the crude extract. However, from the perspective of the pharmaceutical and health care, the purification analysis of glycoprotein is necessary, purified glycoprotein fragments are helpful to clear and specify their functions (Satoshi and Katasumi 1997; Wu et al. 2008; Chen et al. 2012).

The current studies about the chemical compositions of seahorse mainly focus on trace element, amino acids, polypeptides, phospholipids, steroids, etc (Jiang et al. 2007). There are few reports about the preparation, purification, and biological function of seahorse glycoprotein. In this study, the single factor combined with response surface method (RSM) was applied to optimize the extraction condition of seahorse glycoprotein. The method of Sevage in conjunction with ethanol fractional precipitation method were used to purify crude glycoprotein, and the pure glycoprotein was prepared by the method of column chromatography. From these, we developed the preparation process of the target glycoprotein for the study of biological functions.

## Materials and Methods

### Material

The adult dried seahorses (*Hippocampus kuda* Bleeker, totally 15 g) were purchased from a local drugstore, which was originated from aquaculture by Ningbo Yonghe Aquaculture Company (Ningbo, China). DEAE-52 (Whatman, Maidstone, UK), Sephadex G-100 (Pharmacia, New Jersey, USA), MwCo3500D (Sigma, San Francisco, CA), and other chemicals of analytical reagent were purchased from the Sinopharm Chemical Reagent Co. Ltd (Shanghai, China).

### Pretreatment

The cleaned seahorses were placed into the vacuum-freezing dryer (Freezone12; Labconco Co. Ltd, Kansas City, MO) under −40°C for 48–72 h. The freeze-dried material was then crushed with a portable grinder (DFT-50A, Wenlin, Zhejiang Province, China). The seahorse powder was then degreased by supercritical CO_2_ fluid extraction (SFE-CO_2_) (Speed, ASI Co. Ltd, Allentown, PA), the conditions were CO_2_ pressure of 35 MPa, extracting time of 135 min (including dynamic extraction 10 min + static extraction 125 min), temperature of 58°C and CO_2_ flow rate of 15 L/h (Jiang et al. 2013).

### Determination of glycoprotein yield

To fully reflect the effect of glycoprotein extracting, the content of saccharide and protein in seahorse glycoprotein was determined, respectively, in this study (Li et al. 2012).

### Determination of saccharide content in glycoprotein

**Figure 1:**
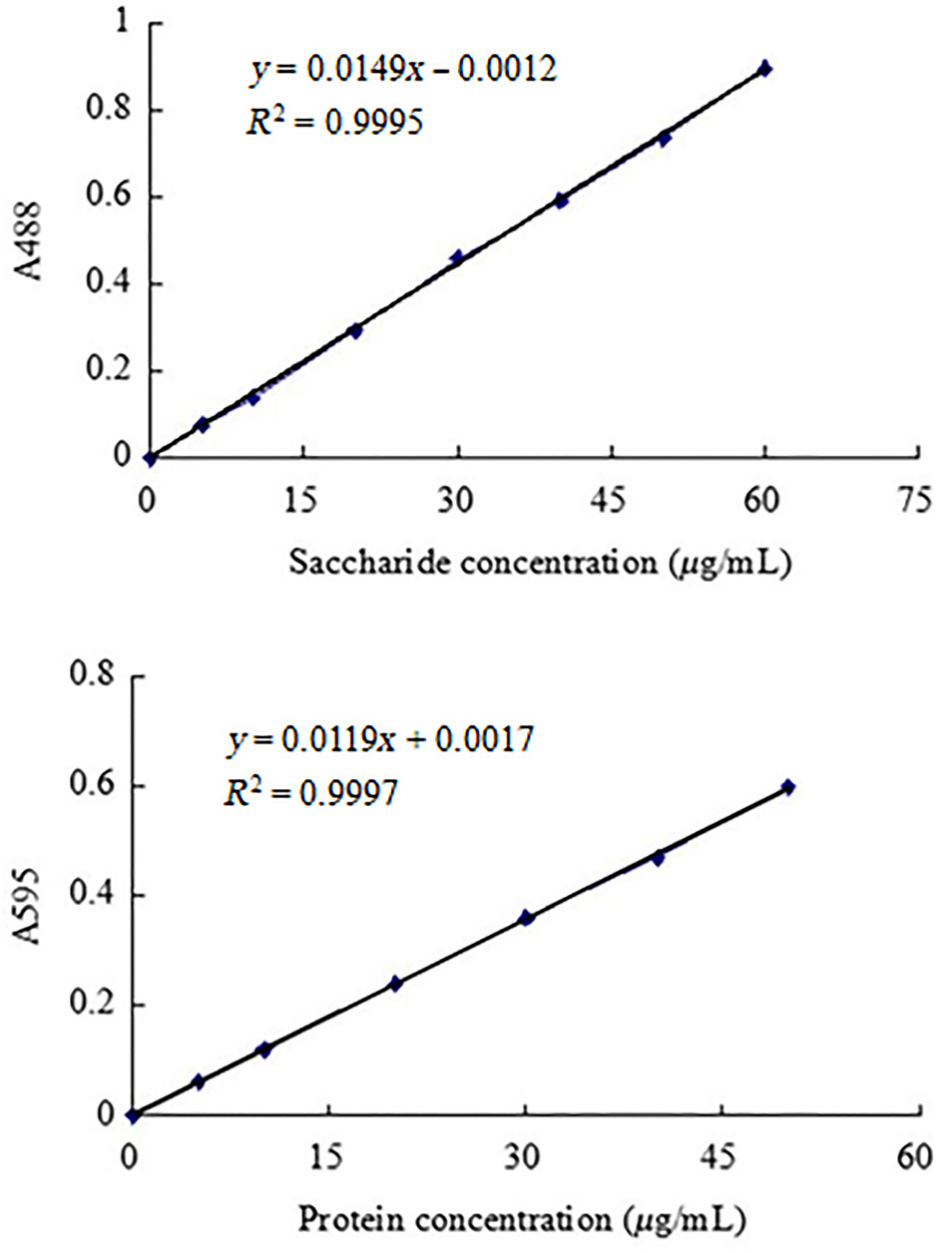
Standard curve of Glucose and Bovine serum albumin for saccharide and protein.

The total saccharide content was determined by the phenol–sulfuric acid method, glucose was used as the standard material, and the results were then expressed as glucose equivalents. 0.1 mg/mL glucose standard solution was taken 0, 0.1, 0.2, 0.4, 0.6, 0.8, 1.0, 1.2, 1.4, 1.6 mL, and distilled water added to 2 mL, then 1 mL 5% phenol solution was added and the solution well mixed by shaking the tube. Finally, 5-mL concentrated sulfuric acid was added rapidly along the colorimetric tube wall so that the acid can flow evenly along the wall. The solution was left alone for 30 min at room temperature for adequate response. The reaction solution in the appropriate amount was scanned under 200–600 nm (Meipuda UV-3300, Shanghai, China) to determine the maximum absorption wavelength of saccharide components, and distilled water was used as the blank sample. The result of the maximum absorption wavelength is 488 nm. Therefore, the above samples were measured at 488 nm, and then the standard curve of glucose was drawn, based on the regression equation *y* = 0.7434*x* − 0.0012, *R*^2^ = 0.9995, where *x* is the concentration of saccharide (*μ*g/mL), *y* is the absorbance value. The sample solution was diluted to an appropriate density and measured by the same method, and then the saccharide content was obtained by the glucose standard curve. The glucose standard curve is shown in Figure1.

### Determination of protein content in glycoprotein

The protein content was determined by the Coomassie Brilliant Blue method, bovine serum albumin (BSA) was used as standard protein, and the results were then expressed as protein equivalents. 0.1 g Coomassie Brilliant Blue G-250 was dissolved in 50 mL 95% ethanol, then 100 mL 85% phosphoric acid was added, before the distilled water was added to 1000 mL. The prepared Coomassie Brilliant Blue G-250 was filtered and stored with a brown bottle for use. 0.1 mg/mL BSA standard solution was taken 0, 0.1, 0.2, 0.4, 0.6, 0.8, 1.0 mL, and distilled water was added to 1 mL, then 1 mL 5% phenol solution was added, before 5 mL Coomassie Brilliant Blue G-250 was added along the colorimetric tube wall and mixed well by shaking the tube. The solution was left alone for 10 min at room temperature for adequate response. The above samples were measured at 595 nm, and then the standard curve of BSA was drawn, based on the regression equation *y* = 0.5932*x* + 0.0017, *R*^2^ = 0.9997, where *x* is the concentration of protein (*μ*g/mL), *y* is the absorbance value. The sample solution was diluted to an appropriate density and measured by the same method, and then the protein content was obtained by the BSA standard curve. The BSA standard curve is shown in Figure1.

#### Preparation of seahorse glycoprotein

##### Single-factor experiment

To ensure the activity of seahorse glycoprotein and to avoid original structure damage of glycoprotein from acid, alkali, and hydrolysis, this experiment selected dilute brine formulation to extract glycoprotein and supplemented by ultrasonic treatment to improve the rate of glycoprotein. The pretreated seahorse dry powder (0.2 g) was extracted in an Erlenmeyer flask, the extracting conditions were analyzed, such as extracting time, salt concentration, extracting temperature, raw material, and water ratio. Each factor and its gradients were as follows: extracting time of 1, 2, 3, 4, 5, 6 h; salt concentration of 0, 0.05, 0.10, 0.15, 0.20, 0.25 mol/L; extracting temperature of 30°C, 40°C, 50°C, 60°C, 70°C, 80°C; raw material and water ratio of 1:10, 1:15, 1:20, 1:25, 1:30, 1:35. Each gradient of a factor had three replicates. The first step of experiment is ultrasonic-assisted extraction, ultrasonic power is 500 W, ultrasonic time is 20 min, and then the sample was placed in a water bath to extract, shook once every 30 min. After the completion of the extraction, the sample was filtered through filter paper and set the volume for next step.

##### Response surface process optimization

The software Design Expert V7.0.0 (Stat-Ease, Inc., Minneapolis, MN) was selected to determine the response surface (Central Composite Design) for optimizing the conditions of extraction. The levels of experimental factors are shown in Table1. The absorbance value of saccharide and protein content was measured as response values. By analyzing the relationship among the four factors and response values based on the experiment data, we can obtain the optimum extraction condition of seahorse glycoprotein.

**Table 1:** Coding of factors and levels in the glycoprotein extracting process

Level	Factors			
	Time	Salt	Temperature	Ratio
	A (h)	B (mol/L)	C (°C)	D (mL/g)
1	3	0	60	1:15
0	4	0.05	70	1:20
−1	5	0.1	80	1:25

##### Purification of seahorse glycoprotein

The purification process of seahorse glycoprotein is divided into two parts: the crude purification of glycoprotein and ethanol fractional precipitation, and the further purification was separated by column chromatography (Yang et al. 2011). The extracting solution of seahorse was concentrated to an appropriate volume under reduced pressure at 60°C using a rotary evaporator, then treated with Sevage reagent to remove free protein once at 4°C overnight. The volume ratio of the glycoprotein and Sevage reagent is 4:1, chloroform and butanol is 5:1. Glycoprotein layer was collected and purified by ethanol-graded precipitation. Ethanol was, respectively, added to final concentration of 50, 60, 70, 80, 90%, then kept at 4°C for 12 h and the precipitate was collected and washed with acetone and diethyl ether twice. The precipitate was reconstituted with distilled water, then dialyzed with 3500 Da dialysis bag for 72 h, and lyophilized to get the seahorse crude glycoprotein.

##### DEAE-52 column chromatography

The newly acquired DEAE-52 cellulose wadding 50 g was steeped with 10 times of distilled water volume at room temperature for 24 h to full swelling, then repeatedly washed with distilled water and thoroughly stirred, finally the fine particles was decanted after natural subsidence. Next, the cellulose wadding was treated “alkali, acids, alkali” processing with 0.5 mol/L NaOH, 0.5 mol/L HCl, 0.5 mol/L NaOH and subsided naturally after sufficiently stirring, then the upper acid-alkali solution was decanted and repeatedly washed with distilled water to neutral (pH = 7.0) before dealing with the next step. Eventually, the DEAE-52 cellulose wadding was washed to neutral and treated with wet packing column (ion exchange column specification (1.6 × 50 cm). The column was stood overnight and balanced with distilled water for 24 h. 0.2 g of the crude glycoprotein was dissolved in 5 mL of deionized water to configure to 40 mg/mL solution of glycoprotein, and the sample solution was filtrated through 0.5 mm pinhole membrane. First, the eluent and elution mode of DEAE-52 column chromatography were determined. The elution rate is 1.0 mL/min controlled by current pump (Puxi HL-2, Shanghai, China), 5 mL/tube collected by automatic fraction collector (Puxi BSZ - 100, Shanghai, China). Throughout the experiment, the UV detector (Puxi HD-21-2, Shanghai, China) connecting with chromatography equipment detected elution profile of the sample at 280 nm wavelength. Every tube was measured the absorbance value of sugar at 488 nm wavelength by phenol–sulfuric acid and protein at 280 nm wavelength by UV absorption. Overlapping peaks part of sugar and protein were collected and dialyzed with 3500 Da dialysis bag for 72 h, and lyophilized and preserved.

1. Selection of eluent: The sample solution was, respectively, eluted with 0–2 mol/L NaCl and 0–1 mol/L NaHCO_3_ by the mode of gradient elution. The absorbance value of saccharide and protein of every eluent was measured to construct the elution profile.
2. Selection of elution mode: The sample solution was, respectively, eluted with 0–1 mol/L NaHCO_3_ by the mode of gradient elution and 0.05, 0.1, 0.5 mol/L NaHCO_3_ by the mode of phased elution. The absorbance value of saccharide and protein of every eluent was measured to construct the elution profile.

##### Sephadex G-100 column chromatography

The purchased Sephadex G-100 10 g was steeped with 20 volumes of distilled water at room temperature for 24 h to full swelling, then repeatedly washed with distilled water and thoroughly stirred, finally the fine particles were decanted after natural subsidence. Next, the cellulose wadding was treated with 0.2 mol/L NaOH and subsided naturally after sufficiently stirring, and then the upper alkali solution was decanted and repeatedly washed with distilled water to neutral. Before packing, the filler was boiled to remove bubbles and to have a bactericidal effect. Finally, the Sephadex G-100 was treated with wet packing column (ion exchange column specification (1.6 × 50 cm). The column stood overnight and balanced with distilled water for 24 h. 0.02 g of the glycoprotein sample was dissolved in 2-mL deionized water to configure to form 10 mg/mL solution of glycoprotein, and the sample solution was filtrated through 0.5-mm pinhole membrane. The sample solution was eluted by distilled water, the elution rate is 1.0 mL/min, 5 mL/tube. Throughout the experiment, the UV detector connecting with chromatography equipment detected elution profile of the sample at 280 nm wavelength. Every tube was measured the absorbance value of sugar at 488 nm wavelength by phenol-sulfuric acid and protein at 280 nm wavelength by UV absorption. Overlapping peaks part of saccharide and protein were collected and lyophilized to get the seahorse pure glycoprotein.

##### Data analysis

With the aid of Microsoft Excel, SAS Deployment Wizard 9.2 (SAS Institute Inc., Cary, NC, USA) and Design-Expert 7.0.0 (Stat- Ease, Inc., Minneapolis, MN), the conditions and effects of extraction and purification of the seahorse were analyzed and compared by the one-way ANOVA.

## Results

### Effect of single-factor experiment on glycoprotein extraction

**Figure 2:**
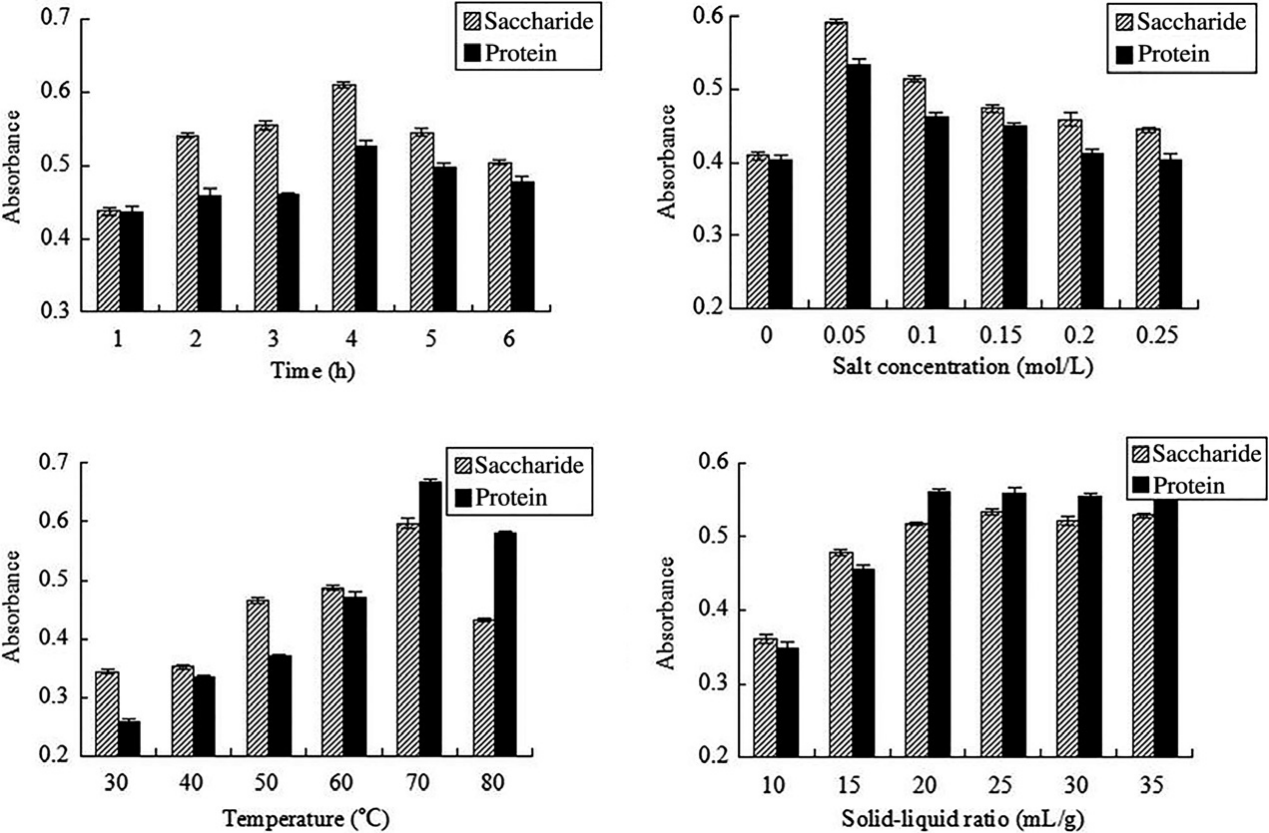
Effect of extraction time, salinity, extraction temperature and solid–liquid ratio of saccharide and protein on seahorse glycoprotein extracting process.

The results of single-factor experiment are shown in Figure2. With the increase in extracting time, saccharide and protein absorption values decreased after an initial increase, and the changing trends were basically the same, when the extracting time was 4 h, saccharide and protein both reached the maximum rate. Similarly, the best salt concentration was 0.05 mol/L, extracting temperature was 70°C. With the increase in liquid–solid ratio, saccharide and protein absorption values were gradually increased, but the changing trends were stable when the liquid–solid ratio was 1:20. Therefore, from the view of extraction effects and saving solvent, the optimal liquid–solid ratio was 1:20.

### Analysis of response surface optimization process

#### Regressive model establishment and significant test

Using Design-Expert 7.0.0 software for response surface central composite design of four factors and three levels, there are 29 groups. The results for saccharide and protein are shown in Table2. The experimental data were analyzed by the following quadratic regression:

**Table 2:** Design and results of the glycoprotein extracting experiment

Run	A: Time	B: Salt	C: Tem	D: Ratio	Saccharide	Protein
1	0	−1	−1	0	0.526 ± 0.03	0.345 ± 0.04
2	−1	0	0	1	0.421 ± 0.02	0.628 ± 0.02
3	0	1	0	−1	0.444 ± 0.04	0.61 ± 0.01
4	0	0	0	0	0.729 ± 0.04	0.65 ± 0.02
5	1	−1	0	0	0.53 ± 0.03	0.494 ± 0.02
6	0	0	1	1	0.469 ± 0.02	0.77 ± 0.01
7	0	−1	0	1	0.454 ± 0.02	0.638 ± 0.05
8	−1	1	0	0	0.459 ± 0.04	0.633 ± 0.04
9	0	0	0	0	0.44 ± 0.03	0.689 ± 0.04
10	0	0	0	0	0.686 ± 0.02	0.638 ± 0.02
11	0	−1	0	−1	0.406 ± 0.02	0.554 ± 0.03
12	−1	0	1	0	0.458 ± 0.02	0.765 ± 0.01
13	−1	0	0	−1	0.459 ± 0.03	0.676 ± 0.03
14	0	0	1	−1	0.452 ± 0.03	0.73 ± 0.03
15	1	0	−1	0	0.651 ± 0.03	0.325 ± 0.01
16	0	0	0	0	0.664 ± 0.03	0.724 ± 0.03
17	0	0	0	0	0.697 ± 0.04	0.678 ± 0.04
18	1	0	1	0	0.525 ± 0.02	0.689 ± 0.02
19	0	1	−1	0	0.697 ± 0.03	0.333 ± 0.03
20	0	−1	1	0	0.421 ± 0.02	0.58 ± 0.05
21	1	0	0	−1	0.455 ± 0.05	0.607 ± 0.02
22	1	0	0	1	0.458 ± 0.02	0.602 ± 0.01
23	0	0	−1	−1	0.525 ± 0.04	0.309 ± 0.02
24	−1	0	−1	0	0.537 ± 0.04	0.378 ± 0.02
25	−1	−1	0	0	0.472 ± 0.01	0.431 ± 0.02
26	0	1	0	1	0.698 ± 0.01	0.626 ± 0.02
27	1	1	0	0	0.653 ± 0.02	0.691 ± 0.03
28	0	1	1	0	0.627 ± 0.04	0.578 ± 0.04
29	0	0	−1	1	0.616 ± 0.02	0.378 ± 0.04



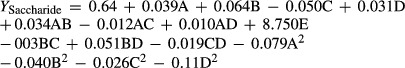


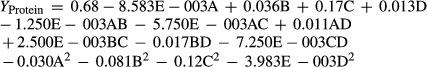


where, A, B, C, and D were extracting time, salt concentration, extracting temperature, raw material, and water ratio, respectively. AB, AC, AD, BC, BD, and CD were the interactions. The response surface models' *P*-value Prob > *F* of saccharide and protein were significant (0.039 and 0.002, respectively). Lack of fit reported the probability difference between the predicted value and the actual measured value, whose *P*-value Prob > *F* values were, respectively, 0.9728 and 0.0671. Therefore, we could use the two models for optimizing the extraction process on seahorse glycoprotein.

#### Analysis of response surface

**Figure 3:**
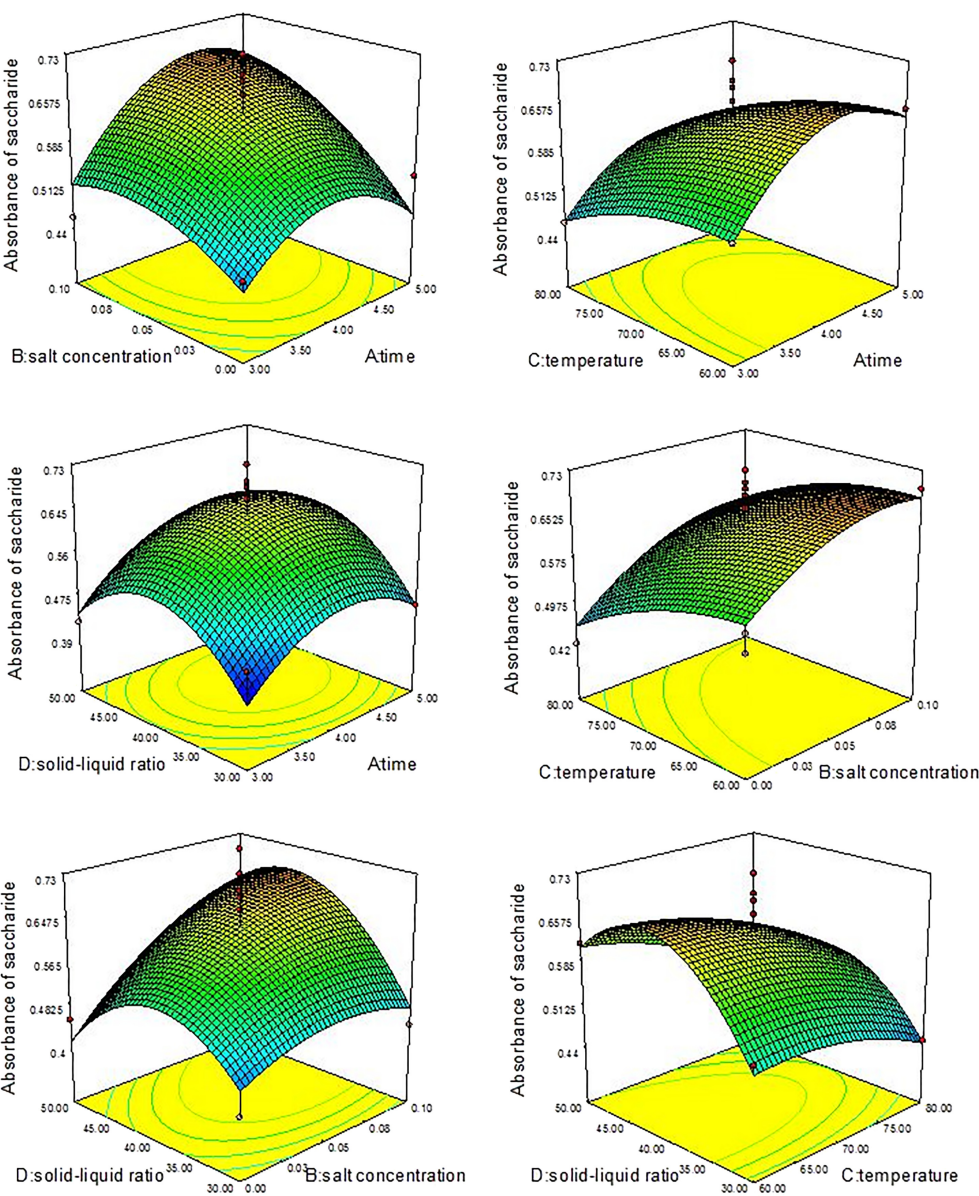
The interactions among time, salt concentration, temperature, and solid–liquid ratio value in response surface experiment of saccharide.

**Figure 4:**
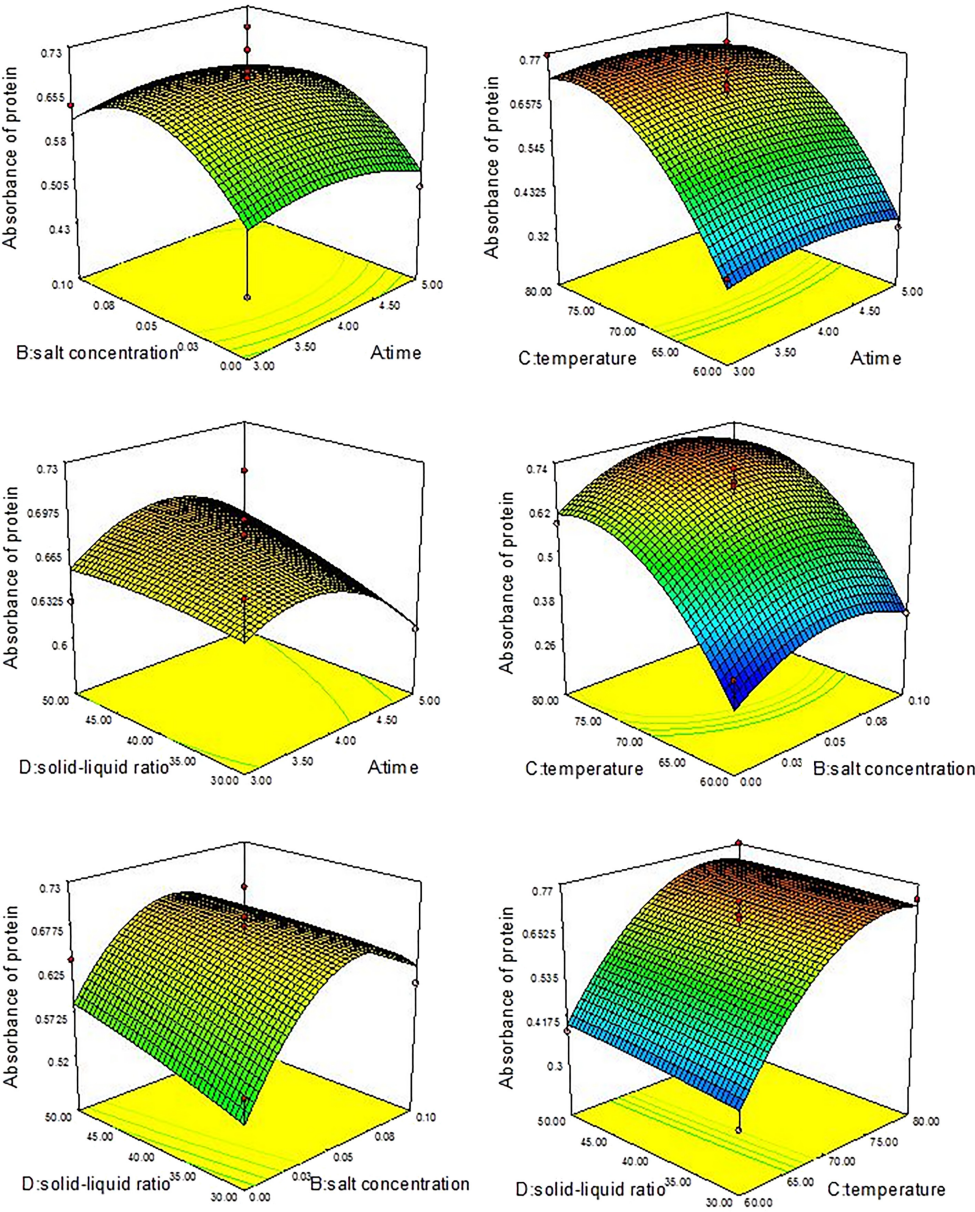
The interactions among time, salt concentration, temperature, and solid–liquid ratio value in response surface experiment of protein.

According to the regression equations of *Y*_Saccharide_ and *Y*_Protein_, Table3, Figure3, and Figure4, we found that the factors B, C, A^2^ (*P*-value Prob > *F* values <0.05), and D^2^ had a significant influence (*P*-value Prob > *F* values < 0.01) on the saccharide extraction process, but interactions had no significant (*P* > 0.05) influence. On the saccharide extraction process, the factors C, B^2^, C^2^ had a significant influence (*P*-value Prob > *F* values < 0.01), but interactions also had no significant influence (*P* > 0.05). *F* value of each factor reflects the importance of experiment targets. The greater the *F* value, the greater impact on the whole experiment targets. As is shown in Table3, the *F* values of saccharide affected by each factor are: *F*(Time) = 2.99, *F*(Salt) = 8.14, *F*(Tem) = 4.95, *F*(Ratio) = 1.93, that is, salt concentration > extracting temperature > extracting time > raw material and water ratio; The *F* values of protein affected by each factor are: *F*(Time) = 0.20, *F*(Salt) = 3.45, *F*(Tem) = 78.40, *F*(Ratio) = 0.46, that is, extracting temperature > salt concentration > raw material and water ratio > extracting time.

**Table 3:** ANOVA results of sugar and protein from seahorse for the yield of chondroitin sulfate

Source	Sum of squares		df	Mean square		*F* Value		*P*-value Prob > *F*	
	Saccharide	Protein		Saccharide	Protein	Saccharide	Protein	Saccharide	Protein
Model	0.23	0.49	14	0.016	0.035	2.65	7.94	0.039	0.0002
A-Time	0.018	8.841E-004	1	0.018	8.841E-004	2.99	0.20	0.1.59	0.6623
B-Salt	0.049	0.015	1	0.049	0.015	8.14	3.45	0.0128	0.0843
C-Tem	0.030	0.35	1	0.030	0.35	4.95	78.40	0.0430	<0.0001
D-Ratio	0.012	2.028E-003	1	0.012	2.028E-003	1.93	0.46	0.1859	0.5102
AB	4.624E-003	6.250E-006	1	4.624E-003	6.250E-006	0.76	1.407E-003	0.3970	0.9706
AC	5.522E-004	1.323E-004	1	5.522E-004	1.323E-004	0.091	0.030	0.7671	0.8655
AD	4.202E-004	4.622E-004	1	4.202E-004	4.622E-004	0.069	0.10	0.7961	0.7517
BC	3.062E-004	2.500E-005	1	3.062E-004	2.500E-005	0.051	5.629E-003	0.8253	0.9413
BD	0.011	1.156E-003	1	0.011	1.156E-003	1.75	0.26	0.2069	0.6179
CD	1.369E-003	2.103E-004	1	1.369E-003	2.103E-004	0.23	0.047	0.6418	0.8309
A2	0.041	5.880E-003	1	0.041	5.880E-003	6.69	1.32	0.0215	0.2691
B2	0.011	0.042	1	0.011	0.042	1.75	9.55	0.2070	0.0080
C2	4.320E-003	0.097	1	4.320E-003	0.097	0.71	21.91	0.4125	0.0004
D2	0.074	1.029E-004	1	0.074	1.029E-004	12.19	0.023	0.0036	0.8812
Residual	0.085	0.062	14	6.057E-003	4.441E-003				
Lack of Fit	0.031	0.058	10	3.099E-003	5.758E-003	0.23	5.01	0.9728	0.0671
Pure Error	0.054	4.597E-003	4	0.013	1.149E-003				
Cor Total	0.31	0.56	28						

The value of Prob > *F* (a) represents difference between the model and each factor; when it is >0.05, there is no significant effect; when it is <0.05, there is a difference or effect, and <0.01 means a significant effect.

#### Optimization of extraction condition and verification

When the saccharide and protein yield, respectively, achieving optimal value with Design-Expert 7.0.0, we can attain the optimal parameters as follows: extracting time 4.27 h, salt concentration 0.08 mol/L, extracting temperature 72.77°C, raw material, and water ratio 1:21.41. The theoretical yield of saccharide is 1.129%; the theoretical yield of protein is 5.908%. Combined with the actual operation, the optimum extraction parameters are as follows: extracting time 4.3 h, salt concentration 0.08 mol/L, extracting temperature 73°C, raw material, and water ratio 1:22. Three verificative tests indicate that the actual yield of saccharide is 1.123% and the actual yield of protein is 5.898%, which show that the regression equation and seahorse glycoprotein extraction have an excellent fit.

### Purification of seahorse glycoprotein

#### The best eluent selection of DEAE-52

**Figure 5:**
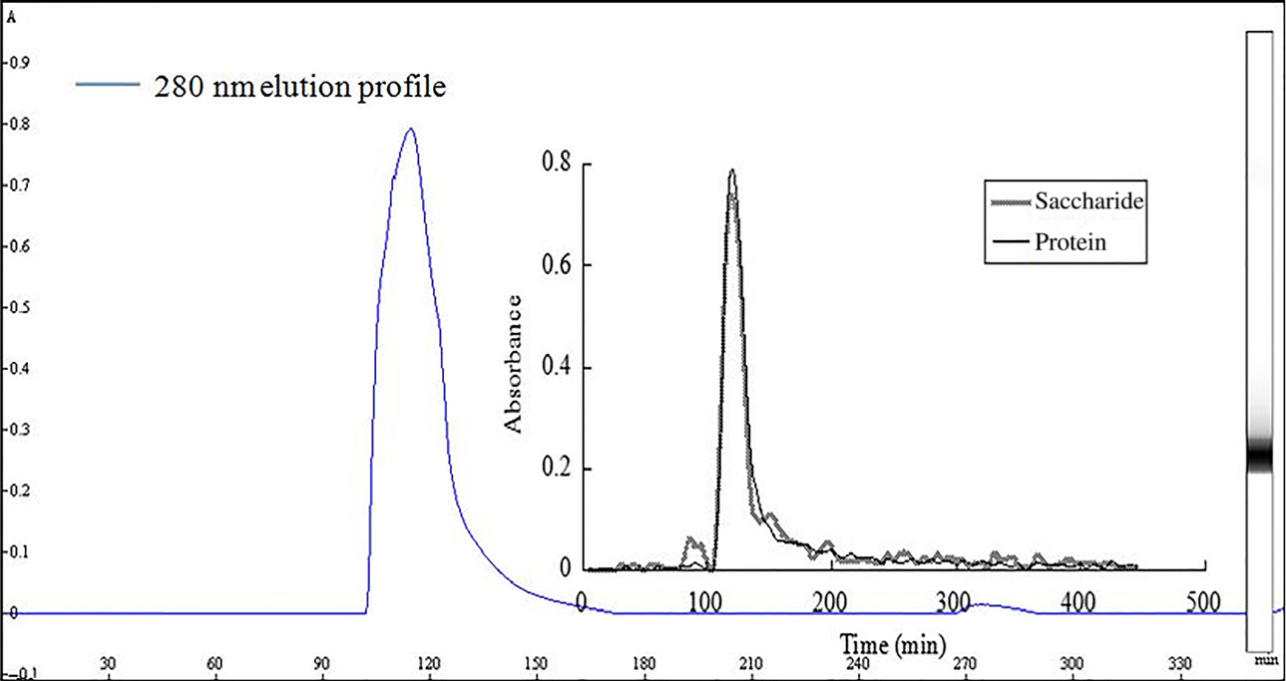
The curve of NaCl gradient elution on DEAE-52 column chromatography.

**Figure 6:**
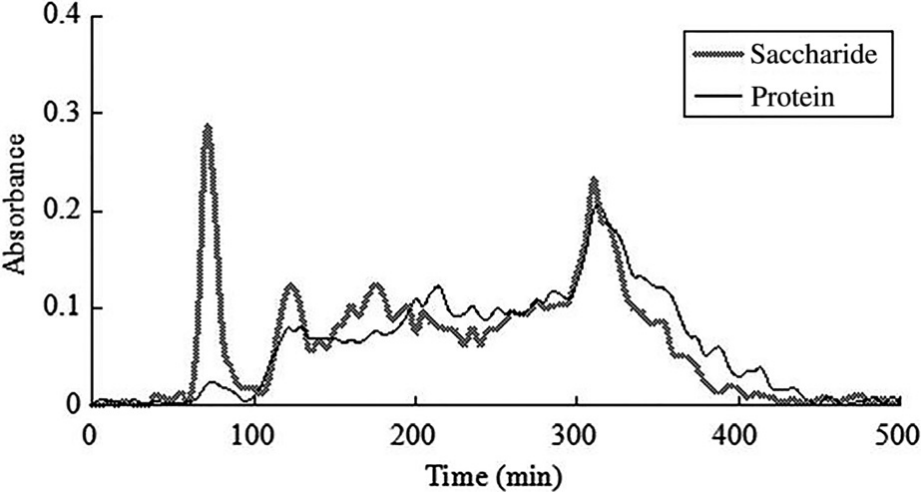
The curve of NaHCO_3_ gradient elution on DEAE-52 column chromatography.

NaCl and NaHCO_3_ were selected to elute seahorse glycoprotein by the mode of gradient elution. The elution profiles are shown in Figure5 and Figure 6. Figure5 is a composite picture of chromatography software image detecting the protein by UV at 280 nm wavelength and glycoprotein image detecting the glycoprotein and protein. As Figure5 shows, when the sample solution was eluted by gradient elution with NaCl, the sample peak time of software image monitored at 280 nm and the protein peak time of glycoprotein image was consistent, which proved that the feasibility of manual detection of seahorse glycoprotein. However, the glycoprotein component substantially flowed from the column at the same time, and it did not achieve the purpose of separation. As Figure 6 shows, when the sample solution was eluted by gradient elution with NaHCO_3_, the glycoprotein component achieved a certain degree of separation, but the peaks were not completely separated. The elution mode needs further improvement.

#### The best elution mode selection of DEAE-52

**Figure 7:**
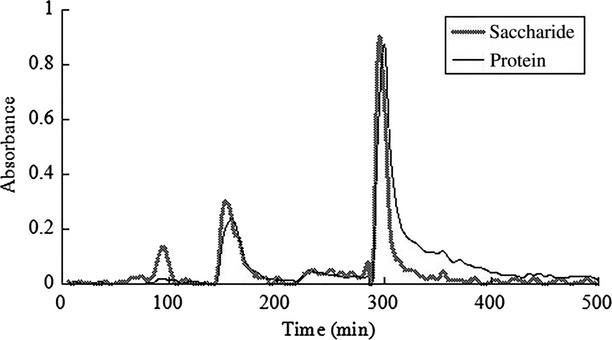
The curve of NaHCO_3_ stage elution on DEAE-52 column chromatography.

As is shown in Figure 7, the glycoprotein component was eluted out three peaks with 0.05, 0.1, and 0.5 mol/L NaHCO_3_ by the mode of phased elution, and the peak patterns were symmetrical. There were totally three elution peaks, but the component of the first elution peak was not glycoprotein, and the component of the other two peaks (HG-1 and HG-2, respectively) was glycoprotein, whose saccharide and protein components both appeared at the same retention time. The two overlapping peak fractions were, respectively, collected and desalted by dialysis, then lyophilized.

#### The result of Sephadex G-100 column chromatography

**Figure 8:**
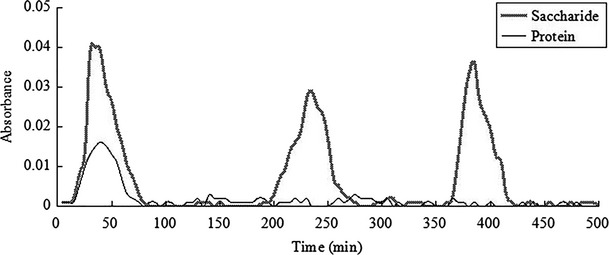
The elution curve of HG-1 by water on G-100 column chromatography.

**Figure 9:**
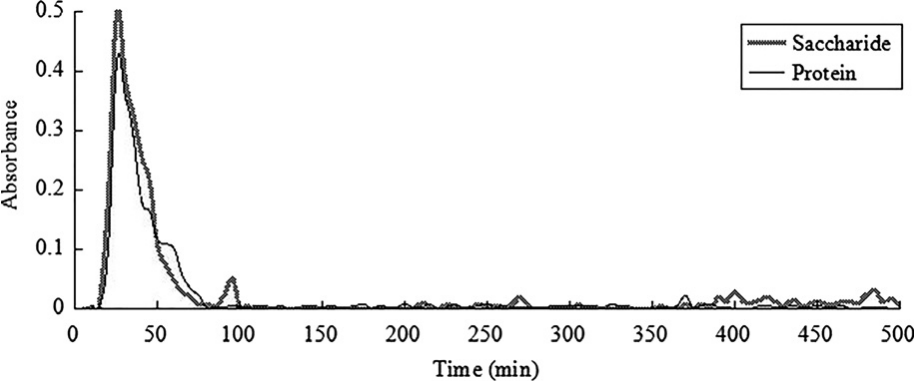
The elution curve of HG-2 by water on G-100 column chromatography.

The purification of DEAE-52 anion exchange chromatography had resulted in two glycoprotein components (HG-1, HG-2), and Sephadex G-100 column chromatography was selected for further purification by distilled water. As is shown in Figure 8, after purification of the HG-1 by Sephadex G-100, a characteristic absorption peak containing both saccharide and protein appeared at 20–115 min and was named HG-11, and the peak pattern was symmetric and no smearing, which indicated the glycoprotein component was homogeneous; two characteristic absorption peaks appeared at 160–230 min and 420–470 min without protein absorption, so the two peaks were impurities polysaccharide peaks. As is shown in Figure 9, after purification of the HG-2 by Sephadex G-100, a characteristic absorption peak containing both saccharide and protein appeared at 15–75 min and was named HG-21, and the peak pattern was symmetric and no smearing, which indicated the glycoprotein component was homogeneous. HG-11 and HG-21 were selected and lyophilized to obtain pure seahorse glycoprotein. The saccharide content was 56.7975 and 39.479%, the protein content was 30.5475 and 51.747%, respectively.

## Discussion and Conclusions

### Extraction technology

The extraction technology of seahorse glycoprotein was investigated in this study. Using Response Surface Methodology combining with ultrasonic assisted extraction, the extraction process was optimized. Ultrasonic extraction technology can cause cell disruption by physical metans to promote the cell contents to flow out, so it can effectively improve the extraction rate (Zou et al. 2011; Cao et al. 2013). Hu et al. (2009) selected the ultrasound-assisted extracting glycoprotein from *Crassostrearivularis crould* and the extraction ratio increased 22.22%. This study selected dilute brine formulation to extract glycoprotein to avoid original structure damage of glycoprotein from acid, alkali, and hydrolysis, and the extracting process was simple and easy to operate (Liu and Zhao 2006). At present, glycoprotein extraction process mainly denominated in the saccharide or protein content for the detection of target, but this cannot fully reflect the overall glycoprotein. Zhang et al. (2012) adopted determination of saccharide content to measure the yield of yam glycoprotein, and the highest extracting rate of yam glycoprotein is 0.74%. In this study, saccharide and protein were, respectively, selected as the detection indicators to conduct common optimization process in order to fully reflect the extracting effect of seahorse glycoprotein, and strive to extract the maximum extent glycoprotein. Based on the above preparation process research of seahorse glycoprotein, we can eventually gain the optimum extraction conditions: ultrasonic time 20 min, extracting time 4.3 h, salt concentration 0.08 mol/L, extracting temperature 73°C, raw material, and water ratio 1:22. The actual yield of saccharide is 1.123% and the actual yield of protein is 5.898%.

### Purification technology

The purification process of seahorse glycoprotein included the crude purification and pure purification. The method of Sevage and ethanol fractional precipitation was chosen for crude purification of glycoprotein. Sevage method deproteinized once and allowed to stand overnight which could effectively remove free protein and avoid the glycoprotein loss causing by too many times of deproteinization. Ethanol-graded precipitation can effectively purify glycoprotein of different molecular weight stages, which can ensure the seahorse glycoprotein integrity (Wu et al. 2008). The column chromatography was used for further purification. Sun et al. (2011) purified crude glycoprotein from Chinese yam tuber with DEAE-52 and Sephadex G-75 column chromatography and obtained two glycoproteins. DEAE-52 anion exchange column purification was performed first, its eluent and elution mode were determined. The seahorse glycoprotein cannot be separated with NaCl eluent. This is probably because NaHCO_3_ eluent can displace the negatively charged protein—acid glycoprotein, which had adsorbed on the cationic groups of cellulose. With the concentration increasing, 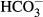 concentration and pH also are increasing, which is more favorable to the elution of acid glycoprotein (Sun et al. 2011). Ultimately, the optimal elution conditions are 0.05, 0.1, and 0.5 mol/L NaHCO_3_ by the mode of phased elution, and overlapping peaks as part of saccharide and protein were further eluted with distilled water by Sephadex G-100 column chromatography. Collected overlapping peak parts of saccharide and protein, we obtained two target glycoprotein HG-11 and HG-21. And the saccharide content was 56.7975 and 39.479%, the protein content was 30.5475% and 51.747%, respectively. The saccharide and protein content were relatively high, which may be related to special effects of the seahorse. The target glycoprotein with a high purity can lay a sound foundation for the study of further structure and function.
